# Adaptive control of 2-wheeled balancing robot by two hemispheric cerebellar neuronal network model

**DOI:** 10.1186/1471-2202-13-S1-P118

**Published:** 2012-07-16

**Authors:** Ruben-Dario Pinzon-Morales, Yohei Ohata, Yutaka Hirata

**Affiliations:** 1 Dept. Computer Science, Chubu University Graduate School of Engineering, Kasugai, Aichi, 487-8501, Japan

## 

Major attention has been given to the cerebellum owing to its principal role during motor learning, sensory-motor transformations, and cognitive functioning. As a result, its neuronal circuitry and learning capabilities have been well identified [1,2], and various mathematical models have been proposed to explain experimental data. Previously we have configured a physio-anatomically inspired cerebellar neuronal network (CNN) controller, and demonstrated that the controller can govern an unstable plant, namely a 2-wheeled balancing robot [3]. The CNN controller followed anatomical cerebellar cortical structure and included granular (Gr), Golgi (Go), basket/stellate (Ba/St), and Purkinje (Pk) cells. Excitatory inputs to the CNN carried by mossy fibers provided the desired motion trajectories. Inhibitory feedback loop between Gr and Go, and feed-forward inhibitory loop between Ba/St and Pk were also included. Inferior olivary (IO) nucleus innervation to Pk via climbing fibers was implemented to convey error signal to the CNN. A proportional and differential (PD) controller sharing the same mossy fiber inputs was introduced to work in tandem while the CNN controller is learning. As such, the error signal via climbing fibers to the Pk was the output of the PD controller. A simple learning rule that adjusts the efficacy of the Gr-Pk synapses was employed to mimic long-term depression and potentiation discovered at this synapse *in vitro* and *in vivo*. Real time simulations and real-world testing evinced that the CNN controller was successful in controlling the unstable plant. Currently a further refinement of the CNN controller was made to enhance its learning capability.

Firstly, exalted by observations of bilateral plasticity during unilateral learning paradigms [4,5], right and left hemispheres of the cerebellum were separately modeled. Secondly the learning rule was modified to include the very low spontaneous spike activity (~ 2 spikes/s) at the climbing fiber input [5,6]. The error signal originated from the concurrent PD controller was split into two by using half-wave rectifiers. The negative and positive waves are fed into right and left hemisphere models, respectively after adding a DC value to mimic the climbing fiber spontaneous firing. Each hemisphere comprises 120 Gr, 1 Go, 6 Ba/St and 1 Pk cell [3]. The desired trajectory tested was a single sinusoid (0.1 Hz) and a band limited (~ 0.3 Hz) noise.

Real time simulation of a 2-wheel balancing robot demonstrated that the proposed two hemispheric CNN not only successfully controlled the robot but also compensated for abrupt perturbations (weight increase) that the PD controller alone cannot manage. Interestingly, in the learning stage, a gradual substitution of the PD output by the CNN controller was observed and eventually the PD output was almost diminished, meaning that the CNN controller successfully took over the PD controller.

## Conclusions

A two hemispheric cerebellar neuronal network controller was constructed and applied to the control of an unstable 2-wheel balancing robot. It was proved that the CNN controller can reproduce a basic form of unilateral learning similar to the real cerebellum. These results are being further evaluated on the real world.
